# ALFF and ReHo Mapping Reveals Different Functional Patterns in Early- and Late-Onset Parkinson’s Disease

**DOI:** 10.3389/fnins.2020.00141

**Published:** 2020-02-25

**Authors:** Yumei Yue, Yasi Jiang, Ting Shen, Jiali Pu, Hsin-Yi Lai, Baorong Zhang

**Affiliations:** ^1^Department of Neurology of the Second Affiliated Hospital, Zhejiang University School of Medicine, Zhejiang University, Hangzhou, China; ^2^Department of Neurology of Sir Run Run Shaw Hospital, Zhejiang University School of Medicine, Zhejiang University, Hangzhou, China; ^3^Department of Neurology of the Second Affiliated Hospital, Interdisciplinary Institute of Neuroscience and Technology, Zhejiang University School of Medicine, Key Laboratory of Medical Neurobiology of Zhejiang Province, Zhejiang University, Hangzhou, China; ^4^Key Laboratory of Biomedical Engineering of Ministry of Education, Qiushi Academy for Advanced Studies, College of Biomedical Engineering and Instrument Science, Zhejiang University, Hangzhou, China

**Keywords:** resting-state fMRI, early-onset Parkinson’s disease, late-onset Parkinson’s disease, motor, vision, emotion

## Abstract

Heterogeneity between late-onset Parkinson’s disease (LOPD) and early-onset Parkinson’s disease (EOPD) is mainly reflected in the following aspects including genetics, disease progression, drug response, clinical manifestation, and neuropathological change. Although many studies have investigated these differences in relation to clinical significance, the functional processing circuits and underlying neural mechanisms have not been entirely understood. In this study, regional homogeneity (ReHo) and amplitude of low-frequency fluctuation (ALFF) maps were used to explore different spontaneous brain activity patterns in EOPD and LOPD patients. Abnormal synchronizations were found in the motor and emotional circuits of the EOPD group, as well as in the motor, emotional, and visual circuits of the LOPD group. EOPD patients showed functional activity change in the visual, emotional and motor circuits, and LOPD patients only showed increased functional activity in the emotional circuits. In summary, the desynchronization process in the LOPD group was relatively strengthened, and the brain areas with changed functional activity in the EOPD group were relatively widespread. The results might point out different impairments in the synchronization and functional activity for EOPD and LOPD patients.

## Introduction

Parkinson’s disease (PD) is a progressive and prevalent neurodegenerative disease in the elderly (de Lau and Breteler, 2006) with several clinical features including motor symptoms (bradykinesia, tremor, rigidity, and gait disturbance) and non-motor symptoms (olfactory dysfunction, constipation, cognitive decline, and depression) (Berg et al., 2015; Postuma et al., 2015). In most cases, the first motor symptom appears after age of 50 (late-onset PD, LOPD), while some patients may present with a parkinsonian syndrome before the age of 50 (early-onset PD, EOPD) (Schrag and Schott, 2006). The clinical features, disease progression and medical management vary between LOPD and EOPD, while little is known about the neural basis.

Recent studies of genetics, disease progression, drug response, and clinical manifestation have provided evidence of different pathological heterogeneity in LOPD and EOPD. Compared with LOPD, EOPD is more affected by genetic factors and the relatives of EOPD patients have a higher risk in developing PD (Stern et al., 1991). Although gait disturbance and dystonia are more common in LOPD patients, EOPD patients have higher prevalence of dyskinesia (Schrag and Schott, 2006; Wickremaratchi et al., 2009), a much slower disease progression (Inzelberg et al., 2004), and levodopa treatment effectiveness (Spica et al., 2013). In terms of non-motor symptoms, EOPD patients present with a better cognitive function and higher incidence of depression (Schrag and Schott, 2006; Spica et al., 2013). Previous studies have found that neuropathological change was different between EOPD and LOPD (Mayer et al., 1986; Gibb and Lees, 1988; Shih et al., 2007; Liu et al., 2015), which may be related to clinical heterogeneity. The pathological results showed that dopaminergic neuron loss of substantia nigra was more serious in EOPD than that in LOPD (Mayer et al., 1986). The studies of single photon emission computed tomography (SPECT) revealed more severe damage to the dopaminergic system and a different dysfunction pattern of striatum in EOPD (Shih et al., 2007; Liu et al., 2015). However, pathological study seems to be limited by the difficulty of obtaining brain specimen and poor repeatability; a more effective tool is needed for further research.

Resting-state functional MRI (rs-fMRI) is a non-invasive neuroimaging technique that has been widely applied to investigate spontaneous brain activity *in vivo* (Smith, 2012). The regional homogeneity (ReHo) and the amplitude of low-frequency fluctuations (ALFFs) are common approaches for depicting regional characteristics of rs-fMRI data. The ReHo calculates the synchronization of low-frequency fluctuations between a given voxel with neighboring voxels (Zang et al., 2004), reflecting the neural function synchronization in local brain region. ALFF measures the ALFFs of individual voxels (Zang et al., 2007), characterizing spontaneous neural activity in local brain region. The rs-fMRI technique has been widely used to explore the neural activity in PD (Lewis et al., 2011; Gao and Wu, 2016; Zhang et al., 2016; Zeng et al., 2017). PD patients showed relatively weakened striatum-cortical and striatum-cerebellar connections and strengthened cortico-cerebellar connection (Wu et al., 2011).

Further to consideration of clinical symptoms, previous studies had found that tremor-dominant PD showed an increased functional connectivity (FC) in the thalamus of the cerebello-thalamo-cortical pathway (Lewis et al., 2011; Zhang et al., 2016) and an increased FC between the subthalamic nucleus (STN) and cerebellum, while PD patients with posture instability and gait difficulty showed a decreased FC among the putamen, pons, and STN (Wang et al., 2016). Default mode network (DMN) dysfunction was involved in cognitive decline (Lucas-Jimenez et al., 2016), and a study found that the efficiency of DMN was lost with the progression of PD (Zeng et al., 2017). Intact prefrontal-limbic connections and reduced amygdala volumes might result in depression in PD patients (Surdhar et al., 2012). These findings indicated that different clinical symptoms were related to specific disrupted brain function patterns. Use of ALFF has revealed significant alterations of brain activity in the motor control related regions (Xiang et al., 2016), visual processing related regions (Yao et al., 2015), and emotional processing related regions (Hu et al., 2015b) in PD. With the ReHo analysis, the dysfunction of neural synchronization in the cortico-striatal-thalamo-cortical circuit (CSTS) (Zhang et al., 2015), visual processing circuit (Li et al., 2016), and emotional processing circuit (Hu et al., 2019) have been clarified in PD. In terms of age of onset, the synchronizations in both the right putamen and left superior frontal gyrus were different between EOPD and LOPD, and the ReHo values of the right putamen were negatively correlated with the Unified Parkinson’s Disease Rating Scale (UPDRS) total scores in LOPD that indicated the distribution of cerebral ReHo was associated with onset-age (Sheng et al., 2016). Sheng et al.’s (2016) study also found higher ReHo values in the left inferior temporal gyrus in EOPD and suggested that further studies are needed to confirm these findings and effects on the emotion regulation in EOPD. In addition, the different striatal FC patterns between EOPD and LOPD were found, and that revealed a more strengthened compensatory mechanism in motor processing network in EOPD (Hou et al., 2016). To our best knowledge, previous studies for different onset-age PD subtypes lack a systemic point of view and integrity; the function change of brain region beyond motor control circuit was not fully explored. To this end, ReHo and ALFF values were calculated to explore the brain activity pattern in motor, visual, and emotional functions for different onset-age PD subtypes.

In the present study, ReHo and ALFF methods were used to evaluate altered spontaneous brain activity pattern in the motor, visual, and emotional circuits for EOPD and LOPD patients compared with age-matched healthy controls (HC). The correlation between these values and clinical features was further analyzed. We assumed that the neural circuits’ change patterns were differently altered in EOPD and LOPD. We supposed to clarify the neural basis mechanism of clinical heterogeneity of EOPD and LOPD.

## Materials and Methods

### Participants

In the current study, 40 patients with idiopathic PD and 20 age-matched HC were enrolled. PD patients were diagnosed according to the UKPD Society Brain Bank criteria (Hughes et al., 1992). The Movement Disorder Society-Unified Parkinson’s Disease Rating Scale (MDS-UPDRS) was used to quantify parkinsonian symptoms. Mini-Mental State Examination (MMSE), Montreal Cognitive Assessment (MoCA), Hamilton Depression Scale (HAMD), and Hamilton Anxiety Scale (HAMA) were conducted to evaluate the cognitive function and emotional status. The inclusion criteria were as follows: age between 40 and 65 years, Hoehn-Yahr stage between 1 and 3, MMSE score ≥ 24, right handed and no history of other neurological disease. All PD patients were sorted into two groups according to their age at onset: 16 EOPD patients (age 49.8 ± 3.6 years, 8 male) and 24 LOPD patients (age 60.8 ± 3.5 years, 16 male), as well as the two groups with age-matched HC, 10 young healthy controls (HCy, age 49.7 ± 2.3 years, 5 male) and 10 older healthy controls (HCo, age 58.0 ± 4.1 years, 3 male), were recruited for further analysis. Anti-parkinsonian medications were withdrawn for 12 h before experiment. The study was approved by the ethics committee of the Second Affiliated Hospital of Zhejiang University School of Medicine, and informed consent was written before experiment.

### Image Acquisition and Analysis

Participants were scanned on a 3T MRI scanner (Magnetom 3T Siemens, Prisma, Germany) with a 20-channel phased array head coil. The anatomical images were obtained by using a volumetric 3D-magnetization prepared rapid acquisition gradient echo sequence (T1WI-3D-MPRAGE) with TR = 2300 ms, TE = 2.32 ms, flip-angle = 8°, slice-thickness = 0.9 mm, and voxel size = 0.9 mm × 0.9 mm × 0.9 mm. The rs-fMRI data were acquired by using an echo-planar image (EPI) pulse sequence with TR = 3000 ms, TE = 26 ms, flip-angle = 76°, slice-thickness = 3 mm, and voxel size = 3 mm × 3 mm × 3 mm.

The image data was analyzed using the brantmaster toolkit^1^ with MATLAB (version 2013a, MathWorks Inc., MA, United States). The preprocessing procedures consisted of the following steps: (1) converting dicom format to nifti with the first 10 time points discarded; (2) time alignment across slices; (3) head motion correction (exclusion criteria: displacement >3 mm or angular rotation >3 in any direction); (4) coregistration and spatial normalization; (5) denoise and band pass filtering (0.01–0.08 Hz); and (6) smoothening (a 6-mm full-width at half-maximum Gaussian kernel). The framewise displacement (FD) value was calculated for head motion estimate (Power et al., 2012). The ALFF map and ReHo map of each subject were calculated with the brantmaster toolkit to evaluate the local spontaneous activity and brain functional synchronization (Zang et al., 2004, 2007). Brain regions with significant difference between EOPD vs HCy or LOPD vs HCo were extracted as masks, signals within masks were calculated using the REST software^2^.

### Statistical Analysis

Demographic and clinical data of EOPD, LOPD, and age-matched HC groups were analyzed using GraphPad Prism software (version 5.0, GraphPad Software, United States). Categorical variables (gender) were analyzed with Pearson Chi-square (χ^2^) tests, and the two sample *t*-test was performed for continuous variables (age, disease duration, H-Y stage, UPDRS, UPDRS-III, MMSE, MoCA, HAMD, and HAMA).

MRI data of EOPD and LOPD with respective age-matched HCs were analyzed and compared using brantmaster toolkit. Two sample *t*-test was used to compare EOPD with HCy, and LOPD with HCo to find the brain regions with significant difference in ALFF and ReHo map. The statistical significance threshold was set at *p* < 0.01, voxel size > 40 corresponding to a corrected *p* < 0.01 as determined by AlphaSim correction. Correlation between clinical data and functional image calculated inside the clusters that showed significant difference was performed using Spearman correlation analysis, *p* < 0.05 was defined as statistical significance with Bonferroni correction. ANOVA analysis for FD value between EOPD, LOPD and corresponding HCs was performed, and *p* < 0.05 was defined as statistically significance.

## Results

### Demographic and Clinical Features

Demographic and clinical features are summarized in Table 1. EOPD and LOPD groups did not statistically differ with respect to disease duration, disease severity and all clinical assessment scale score. The LOPD group showed lower MMSE score (*p* = 0.03, two sample *t*-test) and MOCA score (*p* = 0.003, two sample *t*-test) but higher HAMA score (*p* = 0.01, two sample *t*-test) and HAMD score (*p* = 0.005, two sample *t*-test) as compared with HCo, while there was no significant difference between EOPD and HCy. In addition, the results of mean FD value showed no significant difference between four groups using ANOVA (*p* = 0.27), indicating the head motion characteristics were similar between PD patients and HC.

**TABLE 1:** Demographic information and clinical features of PD patients and healthy controls.

	**EOPD**	**HCy**	**EOPD vs. HCy (*p*-value)**	**LOPD**	**HCo**	**LOPD vs. HCo (*p*-value)**
Sample	14	10		26	10	
Handedness (right)	14	10		26	10	
Age (years)	49.8 ± 3.6	49.7 ± 2.3	0.94	60.8 ± 3.5	58.0 ± 4.1	0.07
Gender (male)	8	5	0.7292	16	3	0.0896
Disease duration (month)	26.9 ± 17.0	NA		22.0 ± 17.5	NA	
H-Y stage	1.71 ± 0.61	NA		1.42 ± 0.58	NA	
UPDRS	34.7 ± 10.6	NA		33.7 ± 14.0	NA	
UPDRS-III	21.1 ± 8.3	NA		19.8 ± 10.5	NA	
MMSE	26.4 ± 4.4	26.4 ± 2.7	0.99	25.9 ± 3.6	27.9 ± 1.3	0.03
MoCA	24.4 ± 4.4	24.6 ± 3.7	0.89	22.0 ± 4.2	26.5 ± 3.2	0.003
HAMD	6.2 ± 4.1	3.7 ± 2.5	0.08	6.7 ± 4.7	3.0 ± 2.5	0.005
HAMA	4.6 ± 2.6	4.1 ± 4.0	0.73	5.8 ± 3.7	3.0 ± 2.2	0.01

*Data are given as mean ± SD. MMSE, Mini-Mental State Examination; MoCA, Montreal Cognitive Assessment; HAMD, Hamilton Depression Scale; HAMA, Hamilton Anxiety Scale; UPDRS, Unified Parkinson’s Disease Rating Scale; NA, not applicable.*

### Altered ReHo in PD Patients

**FIGURE 1:**
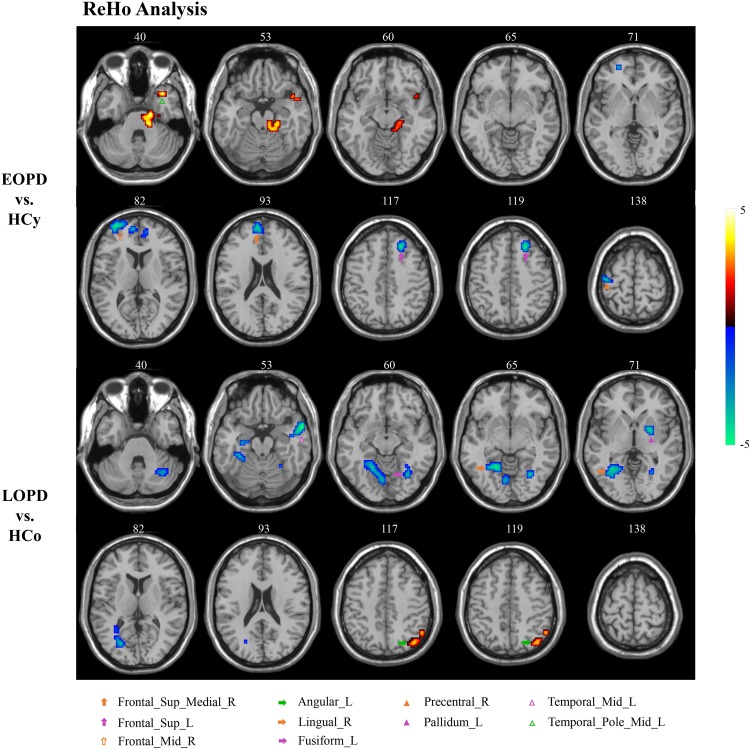
ReHo analysis. Two-sample *t*-tests results are presented, voxel level *p* < 0.01, voxel size >40, corrected by AlphaSim. Area in blue with significantly decreased ReHo value; area in yellow and red with significantly increased ReHo value.

ReHo results of PD subgroups compared to corresponding age-matched HC groups were shown in Figure 1, and the local maxima of ReHo values obtained by two-sample *t*-test are listed in Table 2. For the EOPD compared with HCy, ReHo values of brain regions involved in motor processing circuits changed with a decreased value in right precentral gyrus (PreCG_R) and increased value in left cerebellum_4_5 (lCbe_4_5). Besides, altered ReHo values were also found in emotional processing circuits presented with decreasing in left superior frontal gyrus (SFG_L), right medial superior frontal gyrus (SFGmed_R), right middle frontal gyrus (MFG_R) and left middle temporal_Pole (TPOmid_L). In addition, ReHo values by comparison of LOPD and HCo mainly changed in visual processing circuits including decreased ReHo in bilateral fusiform gyrus (FFG) and right lingual gyrus (LING_R). Decreased ReHo values were also found in the left middle temporal gyrus (MTG_L) and left pallidum (PAL_L). While increased ReHo values were found in left angular gyrus (ANG_L). *p* < 0.01, voxel size >40 was defined as statistically significant corrected by AlphaSim.

**TABLE 2:** ReHo analysis.

**Cluster**	**Brain regions**	**MNI coordinates**			**Voxel size**	**Peak**
		***X***	***Y***	***Z***		
**EOPD vs. HCy**						
Cluster 1	Precentral_R	42	−18	66	62	−3.9154
Cluster 2	Frontal_Sup_L	−21	30	45	41	−4.6176
Cluster 3	Frontal_Sup_Medial_R	6	57	21	94	−4.163
Cluster 4	Frontal_Mid_R	33	60	6	92	−4.8285
Cluster 5		−18	−18	−30	265	5.6964
	Cerebellum_4_5_L				46	
Cluster 6	Temporal_Pole_Mid_L	−36	15	−33	50	5.1976
**LOPD vs. HCo**						
Cluster 1	Fusiform_L	−30	−66	−12	70	−3.9464
Cluster 2	Lingual_R	21	−54	−9	325	−4.6391
	Fusiform_R				80	
Cluster 3	Temporal_Mid_L	−60	6	−21	66	−5.3395
Cluster 4	Pallidum_L	−21	0	−3	44	−4.0632
Cluster 5	Angular_L	−42	−72	42	82	3.7453

*The changed brain regions in fMRI images among EOPD, LOPD, and age-matched HC group. MNI coordinate, Montreal coordinate; the *p*-value is smaller than 0.01 and voxel size is more than 40 (*p* < 0.01, corrected by AlphaSim).*

### Altered ALFF in PD Patients

**FIGURE 2:**
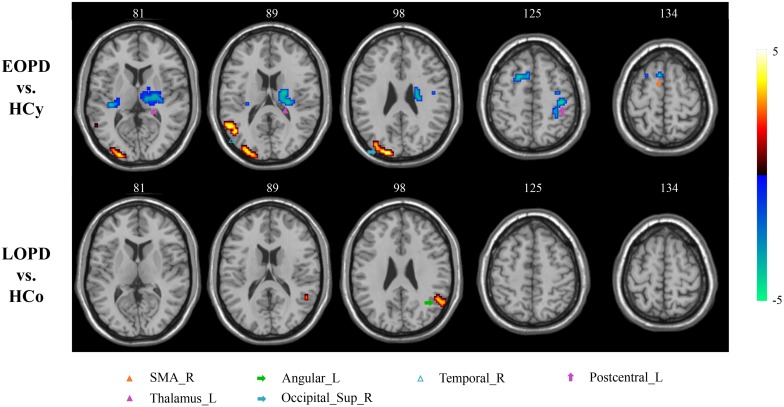
ALFF analysis. Two-sample *t*-test results are presented, voxel level *p* < 0.01, voxel size > 40, corrected by AlphaSim. Area in blue with significantly decreased ALFF value; area in yellow and red with significantly increased ALFF value.

Amplitude of low-frequency fluctuation results of EOPD and LOPD compared with age-matched HC, respectively, are shown in Figure 2, and the local maxima of ALFF values obtained by two-sample *t*-test are listed in Table 3. In comparison with HCy, the EOPD group showed increased ALFF values in the right middle temporal gyrus (MTG_R) related to emotional processing circuits and the areas of visual processing circuits including right superior occipital gyrus (SOG_R) and right middle occipital gyrus (MOG_R), while the decreased ALFF values in the right supplementary motor area (SMA_R) and left thalamus (THA_L) related to motor processing circuit. Additionally, we found increased ALFF values in the left angular gyrus (ANG_L) in the LOPD group compared with the HCo group. Here, *p* < 0.01 and voxel size >40 was defined as statistical significance corrected by AlphaSim.

**TABLE 3:** ALFF analysis.

**Cluster**	**Brain regions**	**MNI coordinates**			**Voxel size**	**Peak**
		***X***	***Y***	***Z***		
**EOPD vs. HCy**						
Cluster 1	Supp_Motor_Area_R	6	12	60	63	−4.7819
Cluster 2	Postcentral_L	−39	−24	51	64	−4.2421
Cluster 3		−30	−27	33	520	−4.3059
	Thalamus_L				48	
Cluster 4	Occipital_Sup_R	24	−93	21	145	4.9508
	Occipital_Mid_R				64	
Cluster 5	Temporal_Mid_R	48	−66	15	42	4.2245
**LOPD vs. HCo**						
Cluster 1	Angular_L	−57	−60	27	63	3.5283

*The changed brain regions in fMRI images among EOPD, LOPD, and age-matched HCs group. MNI coordinate, Montreal coordinate; the *p*-value is smaller than 0.01 and voxel size is more than 40 (*p* < 0.01, corrected by AlphaSim).*

### Significant Correlation Between Functional Image and Clinical Feature

**FIGURE 3:**
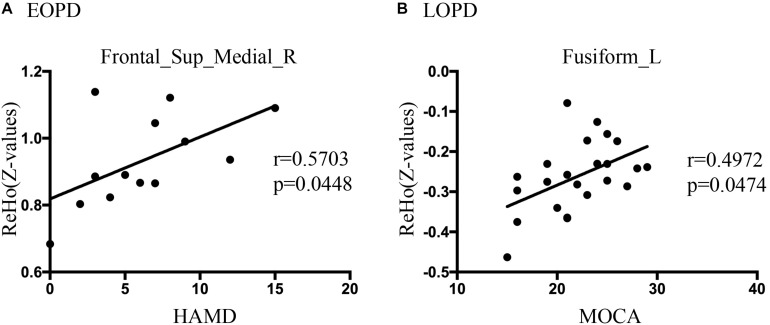
**(A)** Correlations diagram between right medial superior frontal gyrus ReHo values and HAMD score in EOPD group (*r* = 0.5703, *p* = 0.0448, not corrected, Spearman rank correlation). **(B)** Correlations diagram between left fusiform gyrus and MOCA score in LOPD group (*r* = 0.4972, *p* = 0.0474, Bonferroni correction, Spearman rank correlation).

The clinical status with regard to ReHo and ALFF changes were explored using Spearman rank correlations. The results revealed a significantly positive correlation between ReHo values of SFGmed_R and HAMD score in the EOPD group (*r* = 0.5703, *p* = 0.0448, not corrected), shown in Figure 3A. Figure 3B showed a significantly positive correlation between ReHo values of FFG_L and MOCA score in the LOPD group (*r* = 0.4972, *p* = 0.0474, corrected by Bonferroni). There was no significant correlation between ALFF values and clinical features.

## Discussion

The current study exposed the significantly different spontaneous brain activity pattern between EOPD, LOPD and corresponding age-matched HC groups in the motor, emotional, and visual processing circuits. As compared with HCy, EOPD showed lower ReHo and ALFF values in the motor processing circuits, as well as lower ReHo value and higher ALFF value in the emotional processing circuits. As compared with HCo, we found that ReHo decreased in the motor, emotional and visual processing circuits, while ReHo and ALFF increased in the ANG_L related to emotional processing circuit.

### Strengthened Cerebellum Synchronization Might Compensate for Impaired Motor Circuits

Functional connectivity changes in the CSTS circuit have been demonstrated that were associated with clinical manifestations, such as bradykinesia and rigidity (Wu et al., 2011), and weakened FC in the CSTS pathway was found in PD patients (Hacker et al., 2012; Wu et al., 2012). In the current study, EOPD showed decreased ReHo in PreCG_R and decreased ALFF in SMA_R and THA_L, and LOPD showed decreased ReHo in PAL_L. These results indicated both EOPD and LOPD groups have lower synchronization and more impaired functional activity in motor processing circuits. Although the decreased ReHo values of brain areas in this study are different from those reported by Sheng et al.’s (2016) study, all these areas are involved in CSTS circuit. These results accord with the pathological change of substantia nigra results in basal ganglia dysfunction in PD and the oscillatory activity changes in the motor related nuclei (Lindenbach and Bishop, 2013). Additionally, several studies have reported the damage of the dopaminergic system was more serious in EOPD than that in LOPD (Mayer et al., 1986; Schrag and Schott, 2006). Consistent with pathological change, we found a more widespread dysfunction within CSTS circuit in EOPD under the condition of no significantly different disease duration and severity. We also found higher synchronization in the lCbe_4_5 in EOPD. As we know, the cerebellum predominantly contributes to motor coordination, precision, and accurate timing (Fine et al., 2002), and it may also be involved in some cognitive functions (Wolf et al., 2009). Anatomical atrophy in the cerebellum in PD was observed (Lotankar et al., 2017); additionally, functional changes of the cerebellum were also reported. In a prior task-evoked fMRI study, more cerebellum areas were activated, and connections within cortico-cerebellar motor regions were strengthened in PD (Gao et al., 2017) to compensate for basal ganglia deficit when a single tapping task and simple dual-task were performed (Wu et al., 2009, 2011). A rs-fMRI study also described an increased FC in the cerebellum as the more effective compensation mechanism in EOPD as compared with LOPD (Hou et al., 2016). Our results provide additional evidence that strengthened cerebellum synchronization could be a compensation for the striatal dysfunction in EOPD.

### Different Impairments of Visual Processing Circuits in EOPD and LOPD

Visual hallucination, a frequent non-motor symptom of PD (Fenelon et al., 2000; Weil et al., 2016), has been demonstrated to be related to visual circuit dysfunction (Matsui et al., 2006; Arrigo et al., 2017; Hepp et al., 2017). Abnormal brain function was detected even before any visual complaint (Li et al., 2005; Cardoso et al., 2010). Herein, we found different visual circuit disturbances in both PD subgroups; however, no patients presented visual symptoms. EOPD showed greater ALFF in SOG_R and MOG_R than HCy. LOPD showed lower ReHo in FFG and LING_R than HCo. Occipital gyrus, FFG, and LING play important roles in the visual processing circuits (Machielsen et al., 2000; Stoeckel et al., 2009; Weiner and Zilles, 2016). Several studies have reported anatomical atrophy and lower functional activity in FFG, LING, and occipital gyrus in hallucinatory PD than non-hallucinatory PD and HC (Watanabe et al., 2013; Goldman et al., 2014; Yao et al., 2015; Guimaraes et al., 2016). In the current study, we identified the impaired function in FFG and LING in the LOPD group, which may imply that the risk of hallucinations increases with age in LOPD (Fenelon et al., 2000). This phenomenon of presymptomatic image change could be a potential application in clinical diagnosis. Meanwhile, inconsistent with previous studies, EOPD showed an increased functional activity in occipital gyrus, which may suggest a compensation mechanism to sustain visual function in this subgroup.

### EOPD With More Widespread Dysfunction in Emotional Processing Circuits

Emotion disturbance is a common non-motor symptom in PD patients, presenting as apathy, fatigue, anxiety, and depression (Li et al., 2017; Shen et al., 2018). Previous studies indicated that the emotional processing circuits were disrupted in PD patients (Pohl et al., 2017; Bell et al., 2019). In this study, HAMA and HAMD score were significantly higher in LOPD than those in HCo, while there was no significant difference between EOPD and HCy. EOPD showed the ReHo changes in TPOmid_L, SFG_L, SFGmed_R and MFG_R, and ALFF changes in MTG_R. LOPD showed ReHo changes in MTG_L and ANG_L, and ALFF changes in ANG_L. These nuclei located in the frontal lobe and temporal lobe were critically involved in emotional function, and our results indicated that EOPD patients had more widespread dysfunction in emotional processing circuits. Previous studies have identified that hypoactivation or hypometabolism of SFG was associated with fatigue and apathy (Li et al., 2017; Shen et al., 2018; Zhang et al., 2018), as well as that atrophy and dysfunction of MFG might result in depression (Ring et al., 1994; Huang et al., 2016; Chagas et al., 2017; Hanganu et al., 2017) in PD patients. Furthermore, dysfunction within the temporal lobe was also related to depressive symptom (Zeng et al., 2012; Koseki et al., 2013; Kim et al., 2016). An increased FC between the superior temporal gyrus and amygdala was found in PD with depression, which was positively associated with depression severity (Hu et al., 2015a). Atrophy and dysfunction of the temporal pole may be associated with depressive symptoms in PD with depression and major depressive disorder (Feldmann et al., 2008; Zhang et al., 2017). Consistent with this, we found extensive dysfunction of the frontal gyrus, temporal pole, and temporal gyrus in EOPD, which might result in higher incidence of depression.

## Conclusion

In this study, we used ALFF and ReHo analysis to explore the functional abnormality in the motor, visual, and emotional processing circuits between EOPD, LOPD, and age-matched HC, respectively. We found EOPD patients showed different impaired functional activity and synchronization in motor, visual, and emotional processing circuits while LOPD patients only showed impaired synchronization in motor and visual processing circuits, as compared with related age-matched HC. Furthermore, the EOPD group displayed relatively widespread brain areas with changed functional activity, and the LOPD group existed relatively strengthened desynchronization. However, one limitation of this study was a small sample size, which might reduce the statistical power. In addition, long-term use of anti-parkinsonism agents may change the plasticity of the neural circuits of PD patients. Hence, future studies will employ larger sample sizes and investigate the effect of anti-parkinsonism agents in different age of onset subgroups.

## Data Availability Statement

Anonymized data supporting the conclusions of this manuscript will be made available by the authors, without undue reservation, to any qualified researcher.

## Ethics Statement

The studies involving human participants were reviewed and approved by the ethic committee of the Second Affiliated Hospital of Zhejiang University School of Medicine. The patients/participants provided their written informed consent to participate in this study.

## Author Contributions

BZ and H-YL contributed to the conception and design of the study. TS and JP organized the database. YY and YJ performed the statistical analysis. YY wrote the first draft of the manuscript. YJ and TS wrote sections of the manuscript. H-YL modified the manuscript. All authors contributed to the manuscript revision, read, and approved the submitted version.

## Conflict of Interest

The authors declare that the research was conducted in the absence of any commercial or financial relationships that could be construed as a potential conflict of interest.

## Footnotes

1 http://github.com/kbxu/brant

2 http://www.resting-fmri.sourceforge.net

## References

[B1] Arrigo A. Calamuneri A. Milardi D. Mormina E. Rania L. Postorino E. (2017). Visual system involvement in patients with newly diagnosed Parkinson disease. *Radiology* 285 885–895. 10.1148/radiol.2017161732 28696183

[B2] Bell P. T. Gilat M. Shine J. M. McMahon K. L. Lewis S. J. G. Copland D. A. (2019). Neural correlates of emotional valence processing in Parkinson’s disease: dysfunction in the subcortex. *Brain Imaging Behav* 13 189–199. 28812218 10.1007/s11682-017-9754-3

[B3] Berg D. Postuma R. B. Adler C. H. Bloem B. R. Chan P. Dubois B. (2015). MDS research criteria for prodromal Parkinson’s disease. *Mov. Disord.* 30 1600–1611. 10.1002/mds.26431 26474317

[B4] Cardoso E. F. Fregni F. Maia F. M. Melo L. M. Sato J. R. Cruz A. C. (2010). Abnormal visual activation in Parkinson’s disease patients. *Mov. Disord.* 25 1590–1596. 10.1002/mds.23101 20623771

[B5] Chagas M. H. N. Tumas V. Pena-Pereira M. A. Machado-de-Sousa J. P. Carlos Dos Santos A. Sanches R. F. (2017). Neuroimaging of major depression in Parkinson’s disease: cortical thickness, cortical and subcortical volume, and spectroscopy findings. *J. Psychiatr. Res.* 90 40–45. 10.1016/j.jpsychires.2017.02.010 28222355

[B6] de Lau L. M. Breteler M. M. (2006). Epidemiology of Parkinson’s disease. *Lancet Neurol.* 5 525–535. 10.1016/S1474-4422(06)70471-9 16713924

[B7] Feldmann A. Illes Z. Kosztolanyi P. Illes E. Mike A. Kover F. (2008). Morphometric changes of gray matter in Parkinson’s disease with depression: a voxel-based morphometry study. *Mov. Disord.* 23 42–46. 10.1002/mds.21765 17973326

[B8] Fenelon G. Mahieux F. Huon R. Ziegler M. (2000). Hallucinations in Parkinson’s disease: prevalence, phenomenology and risk factors. *Brain* 123(Pt 4) 733–745. 10.1093/brain/123.4.733 10734005

[B9] Fine E. J. Ionita C. C. Lohr L. (2002). The history of the development of the cerebellar examination. *Semin. Neurol.* 22 375–384. 10.1055/s-2002-36759 12539058

[B10] Gao L. Zhang J. Hou Y. Hallett M. Chan P. Wu T. (2017). The cerebellum in dual-task performance in Parkinson’s disease. *Sci. Rep.* 7:45662. 10.1038/srep45662 28358358 PMC5372469

[B11] Gao L. L. Wu T. (2016). The study of brain functional connectivity in Parkinson’s disease. *Transl. Neurodegener.* 5:18. 10.1186/s40035-016-0066-0 27800157 PMC5086060

[B12] Gibb W. R. Lees A. J. (1988). A comparison of clinical and pathological features of young- and old-onset Parkinson’s disease. *Neurology* 38 1402–1406. 10.1212/wnl.38.9.1402 3412587

[B13] Goldman J. G. Stebbins G. T. Dinh V. Bernard B. Merkitch D. deToledo-Morrell L. (2014). Visuoperceptive region atrophy independent of cognitive status in patients with Parkinson’s disease with hallucinations. *Brain* 137(Pt 3) 849–859. 10.1093/brain/awt360 24480486 PMC3983409

[B14] Guimaraes R. P. Arci Santos M. C. Dagher A. Campos L. S. Azevedo P. Piovesana L. G. (2016). Pattern of reduced functional connectivity and structural abnormalities in Parkinson’s Disease: an exploratory study. *Front. Neurol.* 7:243. 10.3389/fneur.2016.00243 28133455 PMC5233672

[B15] Hacker C. D. Perlmutter J. S. Criswell S. R. Ances B. M. Snyder A. Z. (2012). Resting state functional connectivity of the striatum in Parkinson’s disease. *Brain* 135(Pt 12) 3699–3711. 10.1093/brain/aws281 23195207 PMC3525055

[B16] Hanganu A. Bruneau M. A. Degroot C. Bedetti C. Mejia-Constain B. Lafontaine A. L. (2017). Depressive symptoms in Parkinson’s disease correlate with cortical atrophy over time. *Brain Cogn.* 111 127–133. 10.1016/j.bandc.2016.11.001 27918935

[B17] Hepp D. H. Foncke E. M. J. Olde Dubbelink K. T. E. van de Berg W. D. J. Berendse H. W. Schoonheim M. M. (2017). Loss of functional connectivity in patients with parkinson disease and visual hallucinations. *Radiology* 285 896–903. 10.1148/radiol.2017170438 28952907

[B18] Hou Y. Yang J. Luo C. Ou R. Song W. Liu W. (2016). Patterns of striatal functional connectivity differ in early and late onset Parkinson’s disease. *J. Neurol.* 263 1993–2003. 10.1007/s00415-016-8211-3 27394147

[B19] Hu J. Xiao C. Y. Gong D. W. Qiu C. Liu W. G. Zhang W. B. (2019). Regional homogeneity analysis of major Parkinson’s disease subtypes based on functional magnetic resonance Imaging. *Neurosci. Lett.* 706 81–87. 10.1016/j.neulet.2019.05.013 31085291

[B20] Hu X. Song X. Yuan Y. Li E. Liu J. Liu W. (2015a). Abnormal functional connectivity of the amygdala is associated with depression in Parkinson’s disease. *Mov. Disord.* 30 238–244. 10.1002/mds.26087 25545969

[B21] Hu X. Song X. P. Li E. F. Liu J. J. Yuan Y. G. Liu W. G. (2015b). Altered resting-state brain activity and connectivity in depressed Parkinson’s disease. *PLoS One* 10:e0131133. 10.1371/journal.pone.0131133 26147571 PMC4492789

[B22] Huang P. Lou Y. Xuan M. Gu Q. Guan X. Xu X. (2016). Cortical abnormalities in Parkinson’s disease patients and relationship to depression: a surface-based morphometry study. *Psychiatry Res. Neuroimaging* 250 24–28. 10.1016/j.pscychresns.2016.03.002 27107157

[B23] Hughes A. J. Daniel S. E. Kilford L. Lees A. J. (1992). Accuracy of clinical diagnosis of idiopathic Parkinson’s disease:a clinico-pathological study of 100 cases. *J. Neurol. Neurosurg. Psychiatry* 55 181–184. 1564476 10.1136/jnnp.55.3.181 PMC1014720

[B24] Inzelberg R. Schecthman E. Paleacu D. Zach L. Bonwitt R. Carasso R. L. (2004). Onset and progression of disease in familial and sporadic Parkinson’s disease. *Am. J. Med. Genet. A* 124A 255–258. 10.1002/ajmg.a.20405 14708097

[B25] Kim S. M. Park S. Y. Kim Y. I. Son Y. D. Chung U. S. Min K. J. (2016). Affective network and default mode network in depressive adolescents with disruptive behaviors. *Neuropsychiatr. Dis. Treat.* 12 49–56. 10.2147/NDT.S95541 26770059 PMC4706123

[B26] Koseki S. Noda T. Yokoyama S. Kunisato Y. Ito D. Suyama H. (2013). The relationship between positive and negative automatic thought and activity in the prefrontal and temporal cortices: a multi-channel near-infrared spectroscopy (NIRS) study. *J. Affect. Disord.* 151 352–359. 10.1016/j.jad.2013.05.067 23829998

[B27] Lewis M. M. Du G. Sen S. Kawaguchi A. Truong Y. Lee S. (2011). Differential involvement of striato- and cerebello-thalamo-cortical pathways in tremor- and akinetic/rigid-predominant Parkinson’s disease. *Neuroscience* 177 230–239. 10.1016/j.neuroscience.2010.12.060 21211551 PMC3049982

[B28] Li J. Yuan Y. Wang M. Zhang J. Zhang L. Jiang S. (2017). Alterations in regional homogeneity of resting-state brain activity in fatigue of Parkinson’s disease. *J. Neural Transm. (Vienna)* 124 1187–1195. 10.1007/s00702-017-1748-1 28647831

[B29] Li M. Kuroiwa Y. Wang L. Kamitani T. Omoto S. Hayashi E. (2005). Visual event-related potentials under different interstimulus intervals in Parkinson’s disease: relation to motor disability, WAIS-R, and regional cerebral blood flow. *Parkinsonism Relat. Disord.* 11 209–219. 10.1016/j.parkreldis.2004.11.004 15878581

[B30] Li Y. Liang P. Jia X. Li K. (2016). Abnormal regional homogeneity in Parkinson’s disease: a resting state fMRI study. *Clin. Radiol.* 71 28–34. 10.1016/j.crad.2015.10.006 26628410

[B31] Lindenbach D. Bishop C. (2013). Critical involvement of the motor cortex in the pathophysiology and treatment of Parkinson’s disease. *Neurosci. Biobehav. Rev.* 37(Pt 2) 2737–2750. 10.1016/j.neubiorev.2013.09.008 24113323 PMC3859864

[B32] Liu S. Y. Wu J. J. Zhao J. Huang S. F. Wang Y. X. Ge J. J. (2015). Onset-related subtypes of Parkinson’s disease differ in the patterns of striatal dopaminergic dysfunction: a positron emission tomography study. *Parkinsonism Relat. Disord.* 21 1448–1453. 10.1016/j.parkreldis.2015.10.017 26559130

[B33] Lotankar S. Prabhavalkar K. S. Bhatt L. K. (2017). Biomarkers for Parkinson’s disease: recent advancement. *Neurosci. Bull.* 33 585–597. 10.1007/s12264-017-0183-5 28936761 PMC5636742

[B34] Lucas-Jimenez O. Ojeda N. Pena J. Diez-Cirarda M. Cabrera-Zubizarreta A. Gomez-Esteban J. C. (2016). Altered functional connectivity in the default mode network is associated with cognitive impairment and brain anatomical changes in Parkinson’s disease. *Parkinsonism Relat. Disord.* 33 58–64. 10.1016/j.parkreldis.2016.09.012 27659747

[B35] Machielsen W. C. Rombouts S. A. Barkhof F. Scheltens P. Witter M. P. (2000). FMRI of visual encoding: reproducibility of activation. *Hum. Brain Mapp.* 9 156–164. 10739366 10.1002/(SICI)1097-0193(200003)9:3<156::AID-HBM4>3.0.CO;2-Q PMC6871840

[B36] Matsui H. Nishinaka K. Oda M. Hara N. Komatsu K. Kubori T. (2006). Hypoperfusion of the visual pathway in parkinsonian patients with visual hallucinations. *Mov. Disord.* 21 2140–2144. 10.1002/mds.21140 17029272

[B37] Mayer J. M. Mikol J. Haguenau M. Dellanave J. Pepin B. (1986). Familial juvenile parkinsonism with multiple systems degenerations. A clinicopathological study. *J. Neurol. Sci.* 72 91–101. 10.1016/0022-510x(86)90038-9 3950653

[B38] Pohl A. Anders S. Chen H. Patel H. J. Heller J. Reetz K. (2017). Impaired emotional mirroring in Parkinson’s disease-a study on brain activation during processing of facial expressions. *Front. Neurol.* 8:682. 10.3389/fneur.2017.00682 29326646 PMC5741645

[B39] Postuma R. B. Berg D. Stern M. Poewe W. Olanow C. W. Oertel W. (2015). MDS clinical diagnostic criteria for Parkinson’s disease. *Mov. Disord.* 30 1591–1601. 10.1002/mds.26424 26474316

[B40] Power J. D. Barnes K. A. Snyder A. Z. Schlaggar B. L. Petersen S. E. (2012). Spurious but systematic correlations in functional connectivity MRI networks arise from subject motion. *Neuroimage* 59 2142–2154. 10.1016/j.neuroimage.2011.10.018 22019881 PMC3254728

[B41] Ring H. A. Bench C. J. Trimble M. R. Brooks D. J. Frackowiak R. S. Dolan R. J. (1994). Depression in Parkinson’s disease. A positron emission study. *Br. J. Psychiatry* 165 333–339. 10.1192/bjp.165.3.333 7994502

[B42] Schrag A. Schott J. M. (2006). Epidemiological, clinical, and genetic characteristics of early-onset parkinsonism. *Lancet Neurol.* 5 355–363. 10.1016/S1474-4422(06)70411-2 16545752

[B43] Shen Y. T. Li J. Y. Yuan Y. S. Wang X. X. Wang M. Wang J. W. (2018). Disrupted amplitude of low-frequency fluctuations and causal connectivity in Parkinson’s disease with apathy. *Neurosci. Lett.* 683 75–81. 10.1016/j.neulet.2018.06.043 29953925

[B44] Sheng K. Fang W. Zhu Y. Shuai G. Zou D. Su M. (2016). Different Alterations of cerebral regional homogeneity in early-onset and late-onset Parkinson’s disease. *Front. Aging Neurosci.* 8:165 10.3389/fnagi.2016.00165 PMC4940400 27462265

[B45] Shih M. C. Franco de Andrade L. A. Amaro E. Jr. Felicio A. C. Ferraz H. B. (2007). Higher nigrostriatal dopamine neuron loss in early than late onset Parkinson’s disease?–a [99mTc]-TRODAT-1 SPECT study. *Mov. Disord.* 22 863–866. 10.1002/mds.21315 17290452

[B46] Smith K. (2012). Brain imaging: fMRI 2.0. *Nature* 484 24–26. 10.1038/484024a 22481337

[B47] Spica V. Pekmezovic T. Svetel M. Kostic V. S. (2013). Prevalence of non-motor symptoms in young-onset versus late-onset Parkinson’s disease. *J. Neurol.* 260 131–137. 10.1007/s00415-012-6600-9 22820720

[B48] Stern M. Dulaney E. Gruber S. B. Golbe L. Bergen M. Hurtig H. (1991). The epidemiology of Parkinson’s disease. A case-control study of young-onset and old-onset patients. *Arch. Neurol.* 48 903–907. 10.1001/archneur.1991.00530210029018 1953412

[B49] Stoeckel C. Gough P. M. Watkins K. E. Devlin J. T. (2009). Supramarginal gyrus involvement in visual word recognition. *Cortex* 45 1091–1096. 10.1016/j.cortex.2008.12.004 19232583 PMC2726132

[B50] Surdhar I. Gee M. Bouchard T. Coupland N. Malykhin N. Camicioli R. (2012). Intact limbic-prefrontal connections and reduced amygdala volumes in Parkinson’s disease with mild depressive symptoms. *Parkinsonism Relat. Disord.* 18 809–813. 22652466 10.1016/j.parkreldis.2012.03.008

[B51] Wang Z. Chen H. Ma H. Ma L. Wu T. Feng T. (2016). Resting-state functional connectivity of subthalamic nucleus in different Parkinson’s disease phenotypes. *J. Neurol. Sci.* 371 137–147. 10.1016/j.jns.2016.10.035 27871435

[B52] Watanabe H. Senda J. Kato S. Ito M. Atsuta N. Hara K. (2013). Cortical and subcortical brain atrophy in Parkinson’s disease with visual hallucination. *Mov. Disord.* 28 1732–1736. 10.1002/mds.25641 24150865

[B53] Weil R. S. Schrag A. E. Warren J. D. Crutch S. J. Lees A. J. Morris H. R. (2016). Visual dysfunction in Parkinson’s disease. *Brain* 139 2827–2843. 10.1093/brain/aww175 27412389 PMC5091042

[B54] Weiner K. S. Zilles K. (2016). The anatomical and functional specialization of the fusiform gyrus. *Neuropsychologia* 83 48–62. 10.1016/j.neuropsychologia.2015.06.033 26119921 PMC4714959

[B55] Wickremaratchi M. M. Ben-Shlomo Y. Morris H. R. (2009). The effect of onset age on the clinical features of Parkinson’s disease. *Eur. J. Neurol.* 16 450–456. 10.1111/j.1468-1331.2008.02514.x 19187262

[B56] Wolf U. Rapoport M. J. Schweizer T. A. (2009). Evaluating the affective component of the cerebellar cognitive affective syndrome. *J. Neuropsychiatry Clin. Neurosci.* 21 245–253. 10.1176/appi.neuropsych.21.3.24510.1176/jnp.2009.21.3.245 19776302

[B57] Wu T. Wang J. Wang C. Hallett M. Zang Y. Wu X. (2012). Basal ganglia circuits changes in Parkinson’s disease patients. *Neurosci. Lett.* 524 55–59. 10.1016/j.neulet.2012.07.012 22813979 PMC4163196

[B58] Wu T. Wang L. Chen Y. Zhao C. Li K. Chan P. (2009). Changes of functional connectivity of the motor network in the resting state in Parkinson’s disease. *Neurosci. Lett.* 460 6–10. 10.1016/j.neulet.2009.05.046 19463891

[B59] Wu T. Wang L. Hallett M. Chen Y. Li K. Chan P. (2011). Effective connectivity of brain networks during self-initiated movement in Parkinson’s disease. *Neuroimage* 55 204–215. 10.1016/j.neuroimage.2010.11.074 21126588

[B60] Xiang J. Jia X. Q. Li H. Z. Qin J. W. Liang P. P. Li K. C. (2016). Altered spontaneous brain activity in cortical and subcortical regions in Parkinson’s disease. *Parkinsons Dis.* 2016:5246021. 10.1155/2016/5246021 27413576 PMC4930823

[B61] Yao N. Pang S. Cheung C. Chang R. S. Lau K. K. Suckling J. (2015). Resting activity in visual and corticostriatal pathways in Parkinson’s disease with hallucinations. *Parkinsonism Relat. Disord.* 21 131–137. 10.1016/j.parkreldis.2014.11.020 25511330

[B62] Zang Y. Jiang T. Lu Y. He Y. Tian L. (2004). Regional homogeneity approach to fMRI data analysis. *Neuroimage* 22 394–400. 10.1016/j.neuroimage.2003.12.030 15110032

[B63] Zang Y. F. He Y. Zhu C. Z. Cao Q. J. Sui M. Q. Liang M. (2007). Altered baseline brain activity in children with ADHD revealed by resting-state functional MRI. *Brain Dev.* 29 83–91. 10.1016/j.braindev.2006.07.002 16919409

[B64] Zeng L. L. Shen H. Liu L. Wang L. Li B. Fang P. (2012). Identifying major depression using whole-brain functional connectivity: a multivariate pattern analysis. *Brain* 135(Pt 5) 1498–1507. 10.1093/brain/aws059 22418737

[B65] Zeng Q. Guan X. Law Yan Lun J. C. F. Shen Z. Guo T. Xuan M. (2017). Longitudinal alterations of local spontaneous brain activity in Parkinson’s disease. *Neurosci. Bull.* 33 501–509. 10.1007/s12264-017-0171-9 28828757 PMC5636738

[B66] Zhang J. Guo Z. Liu X. Jia X. Li J. Li Y. (2017). Abnormal functional connectivity of the posterior cingulate cortex is associated with depressive symptoms in patients with Alzheimer’s disease. *Neuropsychiatr. Dis. Treat.* 13 2589–2598. 10.2147/NDT.S146077 29066900 PMC5644530

[B67] Zhang J. Q. Wei L. Q. Hu X. F. Xie B. Zhang Y. L. Wu G. R. (2015). Akinetic-rigid and tremor-dominant Parkinson’s disease patients show different Patterns of intrinsic brain activity. *Parkinsonism Relat. Disord.* 21 23–30. 10.1016/j.parkreldis.2014.10.017 25465747

[B68] Zhang J. R. Feng T. Hou Y. N. Chan P. Wu T. (2016). Functional connectivity of Vim nucleus in tremor- and akinetic-/rigid-dominant Parkinson’s disease. *CNS Neurosci. Ther.* 22 378–386. 10.1111/cns.12512 26849713 PMC6492863

[B69] Zhang L. Li T. Yuan Y. Tong Q. Jiang S. Wang M. (2018). Brain metabolic correlates of fatigue in Parkinson’s disease: a PET study. *Int. J. Neurosci.* 128 330–336. 10.1080/00207454.2017.1381093 28918694

